# Evidence of Preferential Aluminum Site Loss during Reaction-Induced Dealumination

**DOI:** 10.1021/jacs.4c13212

**Published:** 2024-12-10

**Authors:** Chao Wang, Andreas Brenig, Jun Xu, Feng Deng, Vladimir Paunović, Jeroen A. van Bokhoven

**Affiliations:** † Institute for Chemical and Bioengineering, Department of Chemistry and Applied Biosciences, ETH Zurich, Vladimir-Prelog-Weg 1, 8093 Zurich, Switzerland; ‡ National Center for Magnetic Resonance in Wuhan, State Key Laboratory of Magnetic Resonance and Atomic and Molecular Physics, Wuhan Institute of Physics and Mathematics, Innovation Academy for Precision Measurement Science and Technology, Chinese Academy of Sciences, Wuhan 430071, China; § Paul Scherrer Institute, Center for Energy and Environmental Sciences, PSI, 5232, Villigen, Switzerland

## Abstract

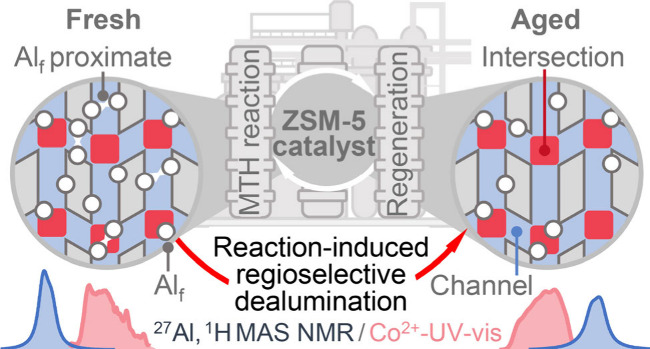

Understanding the mechanism of steam-induced dealumination of zeolite catalysts is of high relevance for tuning their performance and stability in multiple industrial processes. A combination of ^27^Al and ^1^H–^1^H double-quantum single-quantum magic angle spinning nuclear magnetic resonance and diffuse-reflectance ultraviolet–visible spectroscopies identified a preferential dealumination of tetrahedral aluminum sites in H-ZSM-5 zeolites. Framework aluminum atoms facing channels display reactivity toward steam higher than that of those in their intersections. Dealumination randomly occurs on isolated and proximate sites. However, the concentration of the latter sites decays more prominently. These findings contribute to a better understanding of the stability of zeolite catalysts in the presence of steam.

Zeolites are crystalline microporous materials composed of oxygen-sharing SiO_4_ and AlO_4_ tetrahedral (T) units.^1^ The aluminum T sites, also referred to as framework aluminum (Al_F_), induce a negative charge, which, if compensated with protons, creates Brønsted acid sites (BAS). The ability of BAS to activate various molecules, additionally promoted by the confinement effects of the restricted micropore spaces, constitutes the basis for the catalytic applications of zeolites in a broad array of industrially relevant processes.^2−5^ ZSM-5 zeolite, exhibiting the MFI topology with a system of intersecting 10-membered-ring straight and sinusoidal channels of ca. 0.5 nm diameter, is one of the most versatile zeolite catalysts. In various zeolite-catalyzed transformations, such as methanol-to-hydrocarbon (MTH) conversion and biomass valorization, water is present in the feed or evolves as a reaction byproduct.^2,5−8^ At elevated reaction temperatures, the generated steam exhibits the thermodynamically favored potential to cleave the Al–O–Si bridges, leading to a decomposition of BAS, which is better known as dealumination.^8−15^ Moreover, steaming of zeolites is a frequently used postsynthetic treatment for tuning their acidity and increasing their thermal stability.^3,8,12,14−16^

The cleavage of the Si–O–Al bond is proposed to proceed by water attack on aluminum T sites.^8,13,17^ This leads to the formation of a framework-associated aluminum (Al_FA_) site and a silanol group. The successive repetition of the hydrolytic events eventually yields completely dislodged extraframework aluminum (Al_EF_) sites.^11,12,14^ Under more severe steaming conditions, dealumination of multiple framework sites creates mesopores and may eventually lead to the collapse of the crystal.^18^ While the structures of Al_FA_ and Al_EF_ sites remain incompletely understood, it is known that at least some of those sites behave as Lewis acid sites (LAS) and/or interact with remaining BAS.^11,12,19−23^ Consequently, Al_FA_ and Al_EF_ sites are proposed to either strengthen or neutralize BAS, enhance the adsorption of reactants, and stabilize reaction intermediates.^10,14,22,24−26^ Along with a decrease in BAS concentration, these effects have significant implications for catalysis,^6,10,14,24−27^ which makes the understanding of the steaming processes of high fundamental and practical relevance.

Several experimental studies on steaming of the ZSM-5 zeolite indicated that water–aluminum T site interactions may vary across different T sites, causing their different propensities to dealuminate. The observation of a nonuniform distribution of mesopores in steamed ZSM-5 by microscopic inspection led to the proposal that sites in the straight pores are more resistant to dealumination than those in sinusoidal channels.^18^ The ^27^Al multiple-quantum magic angle spinning nuclear magnetic resonance (^27^Al MQ MAS NMR) spectroscopic study indicated that in ZSM-5 zeolites with low aluminum content dealumination preferentially occurs in the sites facing channel intersections, in contrast to mostly even dealumination of all sites in aluminum-rich materials.^14^ A theoretical analysis revealed that the regioselectivity of the dealumination process is primarily determined by the accessibility and solvation of active aluminum T sites.^8^ Consistently, comprehensive ^1^H–^17^O crosscorrelation and ^1^H–^1^H, ^27^Al–^27^Al, and ^17^O–^17^O autocorrelation NMR experiments supported with theory indicated a more favorable dealumination of proximate aluminum sites upon steaming, which is partially caused by the formation of stable Al_F_–Al_FA_ proximates.^28^ Uncovering the zones of preferential zeolite dealumination offers the basis for the engineering of zeolites with enhanced stability under steaming environments.

To gain insights into the dealumination of the ZSM-5 zeolite by reaction-generated steam, the changes in the local environment of the aluminum T sites were analyzed in a commercial H-ZSM-5 catalyst, exhibiting a moderate Si/Al_F_ ratio of 39 (Figure 1, Figure S1), after its use in two (H-ZSM-5_2c_) and six (H-ZSM-5_6c_) sequential MTH reaction–regeneration cycles. This simulates a typical catalyst operation in an industrial MTH reactor, in which periodic regeneration treatments are required to remove the coke deposits and thus restore the catalyst activity (Figure S2). 1D ^27^Al MAS NMR spectra of H-ZSM-5, H-ZSM-5_2c_, and H-ZSM-5_6c_ catalysts, recorded at an increased magnetic field of 18.8 T, unequivocally show the progressive loss of the integrated intensity of the aluminum T resonance with increasing number of reaction–regeneration cycles, corresponding to 36% in H-ZSM-5_2c_ and 54% in H-ZSM-5_6c_ catalysts relative to the parent material (Figure 1). Consistent with this, the ^29^Si MAS NMR also indicates an increase of Si/Al_F_ ratio to ca. 56, corresponding to the loss of ca. 30% of the Al_F_ sites. Notably, in addition to the intensity, the profile of the aluminum T resonances changes. More specifically, based on 3Q ^27^Al MAS NMR spectra (Figure S3), two spectral components centered at ca. 56 and 54 ppm and one broad component centered at ca. 55 ppm were identified and used for fitting the tetrahedral aluminum resonance in respective 1D ^27^Al MAS NMR spectra. The assignment of aluminum T resonances to specific Al_F_ sites is rather complex, mainly due to strong quadrupolar effects.^29−32^ Still, the broad component at 55 ppm can be associated with distorted Al_F_ sites, which is consistent with its low contribution in the parent material and its increasing contribution in dealuminated catalysts. Regarding the two narrower components, several detailed ^27^Al 3Q MAS NMR studies of ZSM-5 materials synthesized with varying structure-directing agents and/or counter cations that steer different aluminum distributions across available T sites, corroborated by characterization by diffuse-reflectance ultraviolet–visible (DR UV–vis) spectroscopy of extraframework Co^2+^ ions,^33^ probe reactions sensitive to Al_F_ siting,^31,34−36^ and density functional theory calculations,^29,30^ postulated the relationships between the profile of tetrahedral resonance in ^27^Al MAS NMR and Al_F_ distribution.^32^ The component centered at ca. 56 ppm is typically ascribed to Al_F_ facing the straight and sinusoidal pores, while the component at ca. 54 ppm preferentially originates from the Al_F_ sites facing the more spacious zones of the intersections. While both 56 and 54 ppm components decrease in their absolute intensities, the decrease of the former is much more pronounced. As a result, the relative ratio of integrals of these two components (*I*_56 ppm_:*I*_54 ppm_) decreases from a value of ca. 1.2 in H-ZSM-5 to ca. 0.5 in H-ZSM-5_6c_. This result points to a higher dealumination propensity of the sites situated in H-ZSM-5 channels compared with those facing zeolite intersections.

**Figure 1:**
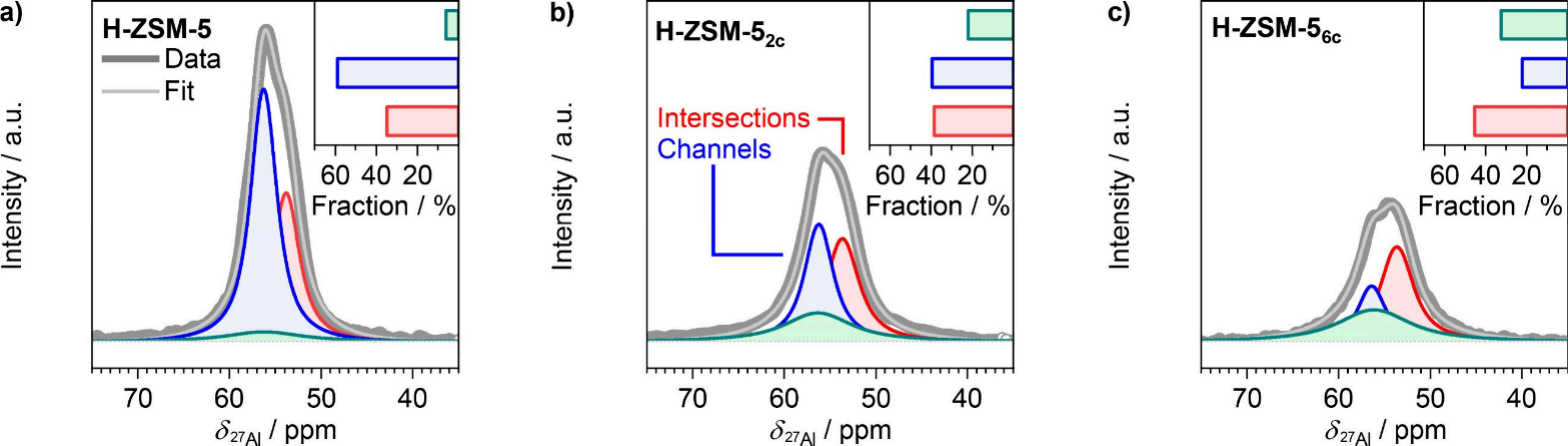
^27^Al MAS NMR spectra (18.8 T) of a) H-ZSM-5, b) H-ZSM-5_2c_, and c) H-ZSM-5_6c_ in the region of the tetrahedral Al_F_ site resonances along with their deconvolution into three components identified based on the ^27^Al 3Q MAS NMR experiment presented in Figure S3. The insets in (a–c) indicate the relative fraction of specific components.

Consistent with the ^27^Al MAS NMR results, the ^1^H MAS NMR signals associated with the BAS protons (3.8 ppm) decrease with an increasing number of the MTH reaction–regeneration cycles, pointing to the progressive dealumination (Figure S4). 2D ^1^H–^1^H DQ-SQ MAS NMR spectroscopy provided additional insights into the proximity between homo- and heteronuclear proton species based on the appearance of auto-(δ_*i*_, 2 × δ_*i*_) and intercorrelation (δ_*i*_, δ_*i*_ + δ_*j*_) peaks (Figure 2, Scheme 1).^20,40^ Notably, in addition to the autocorrelation peak of silanols (1.9, 3.8 ppm) and the intercorrelation peak between these groups and BAS (1.9, 5.8 ppm), the 2D ^1^H–^1^H DQ-SQ MAS NMR spectra of H-ZSM-5 also exhibit an autocorrelation signal of BAS (3.9, 7.8 ppm), pointing to the significant presence of the spatially proximate Al_F_ sites (Figure 2a). In agreement with a loss in overall acidity, the relative intensity of the BAS–BAS peak decreases with an increasing number of MTH reaction–regeneration cycles, as captured by the slices through the SQ dimension (Figure 2b–d). In addition, complementary 2D ^1^H–^1^H DQ-SQ MAS NMR spectra collected at different recoupling times show that the relative intensities of the autocorrelation BAS–BAS peaks exhibit a very similar dependence as a function of recoupling time in parent and aged H-ZSM-5 catalysts (Figures S5–S7). This indicates that during dealumination the probed proximate BAS remain at a similar distance, which is previously estimated to ca. 5 Å,^41^ and that the decrease in the BAS–BAS autocorrelation peak intensity during dealumination stems from a decrease in the concentration of proximate BAS rather than from an increase in average BAS–BAS distance. Taking into account a low concentration of BAS (ca. 2.4 Al_F_ atoms per MFI unit cell), most of the proximate BAS likely exist in the form of pairs. When compared to the relative decrease of the overall BAS peak intensity with respect to H-ZSM-5, the decrease of the autocorrelation BAS–BAS peak is more substantial, following approximately a second-order dependence with respect to the BAS resonance intensity (Figure 2, Figure S8). This is in agreement with the random dealumination model, according to which the concentration of proximate BAS sites is much more affected than the total BAS concentration. Notably, the more substantial loss of proximate Al_F_ sites, and particularly those in the zones of channels, is also suggested by the ^27^Al–^27^Al DQ SQ spectra of H-ZSM-5 and H-ZSM-5_6c_ catalysts (Figure S9).^21,37−39^

**Figure 2:**
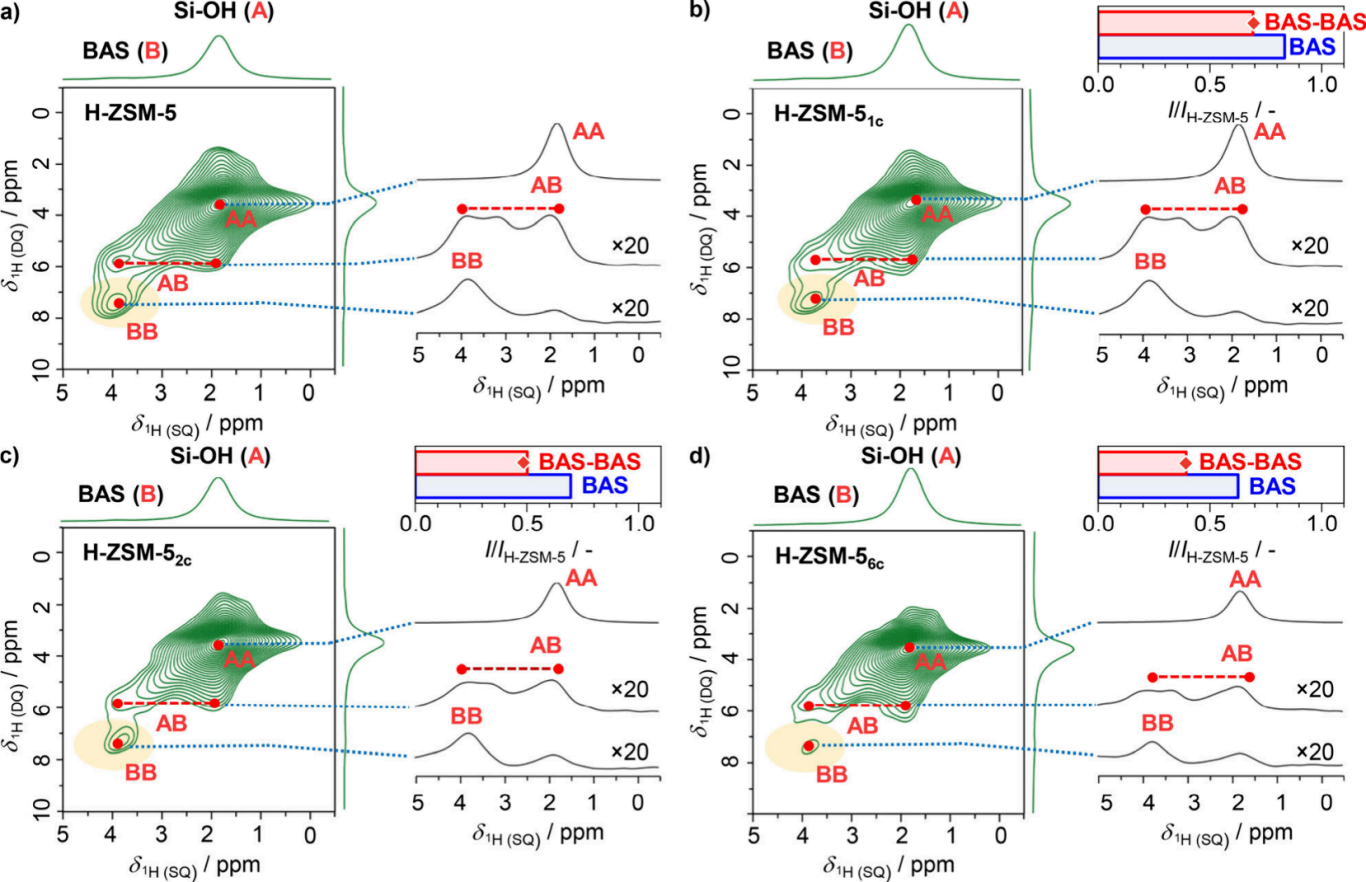
2D ^1^H–^1^H DQ-SQ MAS NMR spectra along with relevant slices of a) H-ZSM-5, b) H-ZSM-5_1c_, c) H-ZSM-5_2c_, and d) H-ZSM-5_6c_ catalysts. The inset bar plots in parts b–d show the relative decrease of the intensity of the BAS-BAS autocorrelation signal in 2D ^1^H–^1^H DQ-SQ MAS NMR spectra and the BAS peak in 1D ^1^H MAS NMR spectra (Figure S4) in corresponding catalysts with respect to those in H-ZSM-5 zeolite. The symbols in the bar plots represent the relative intensity decrease of the BAS–BAS autocorrelation signal, as predicted by the random dealumination model (Figure S8).

**Scheme 1:**
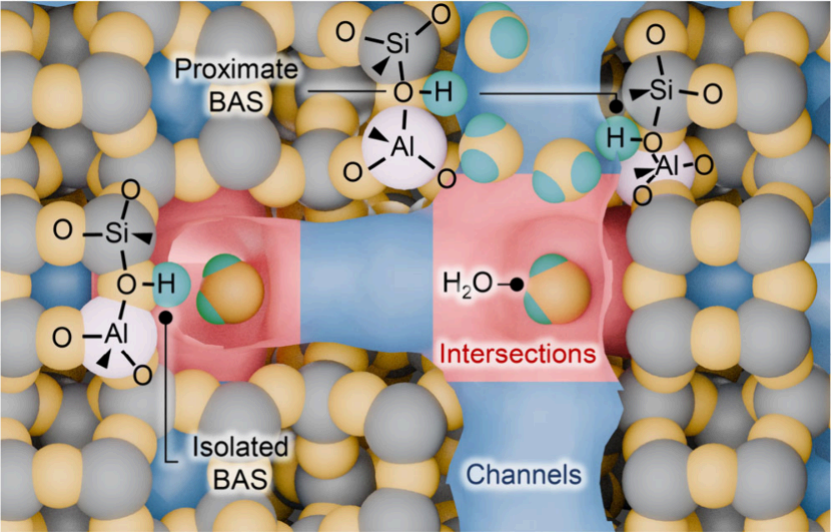
Schematic Representation of Channels and Their Intersecting Regions, Isolated and Proximate BAS in ZSM-5 Zeolite

Site-specific dealumination during the MTH reaction is further investigated by comparing the reactivities of H-ZSM-5 and H-ZSM-5_6c_ catalysts in *n*-hexane and 3-methylpentane (3MP) cracking (Figure 3).^31^ Theoretical and experimental studies indicate that the monomolecular cracking of 3MP involves a bulkier transition state, which is predominantly stabilized in the spacious channel intersections. In contrast, the cracking of *n*-hexane is less sterically demanding, allowing it to proceed in both intersections and channels. Therefore, the ratio of *n*-hexane to 3MP cracking rates, which is better known as the constraint index (CI), provides insights into the aluminum distribution. The cracking of *n*-hexane over H-ZSM-5 proceeds ca. 1.6× faster than 3MP cracking. Despite the apparent loss of BAS, the rate of *n*-hexane cracking over H-ZSM-5_6c_ remains comparable to that of H-ZSM-5, while the rate of 3MP cracking increases. The increase of activity per BAS is attributed to the promoting influence of Al_FA_ or A_EF_ sites on reactant adsorption and transition state stabilization.^10,24−26^ Notably, the cracking rates of the two isomers over the H-ZSM-5_6c_ catalyst are virtually identical. This suggests that in comparison to H-ZSM-5, H-ZSM-5_6c_ contains a lower portion of active sites in the channels than in their intersections.

**Figure 3:**
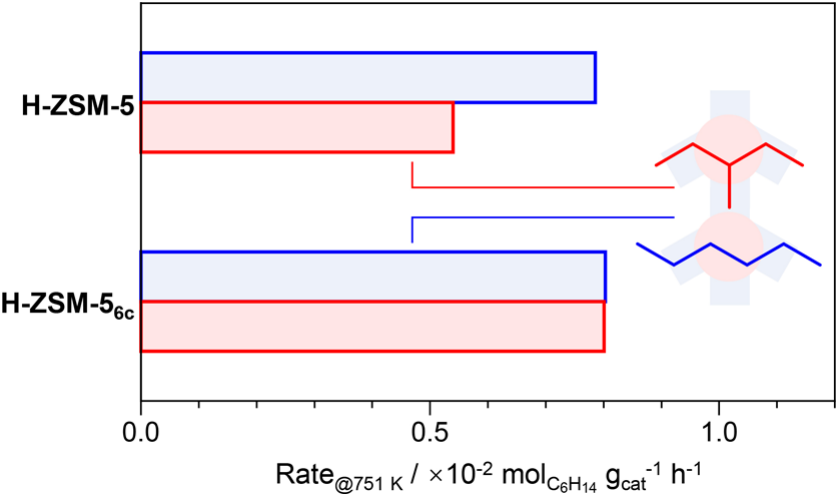
Apparent rates of *n*-hexane and 3-methylpentane (3MP) cracking over the H-ZSM-5 and H-ZSM-5_6c_ catalysts. Conditions: C_6_H_14_:Ar = 4.8:95.2 mol %, WHSV = 16 h^–1^, *T* = 751 K, and *P* = 1.2 bar.

DR UV–vis spectra of H-ZSM-5, H-ZSM-5_2c_, and H-ZSM-5_6c_ catalysts following sequential sodium (Na^+^) and cobalt (Co^2+^) ion exchange and dehydration also support regioselective dealumination (Figure 4). In this approach, extraframework Co^2+^ ions are assumed to bind to two spatially proximate BAS. The *d*–*d* transitions of Co^2+^ ions produce characteristic DR UV–vis spectra in the range of ca. 24 000 to 13 000 cm^–1^, which can be used to analyze the spatial distribution of proximate aluminum sites. Notably, the spectral component centered at around 14 800 cm^–1^, which is ascribed to Co^2+^ sites in the α positions within the straight channels, displays the most prominent decrease in H-ZSM-5_2c_ and H-ZSM-5_6c_ as compared to that of H-ZSM-5 zeolite. In contrast, the relative intensities of the bands at ca. 16 100, 17 300, 18 600, and 21 000 cm^–1^, corresponding to Co^2+^ cations in β positions at the channel intersections, remain mostly unchanged. The contribution of the bands at 20 100 and 21 900 cm^–1^, attributed to Co^2+^ sites in γ positions within the sinusoidal channels and oriented toward intersections, increases. Considering an overall decrease in the Al_F_ site concentration, this result indicates that sites in the straight channels are particularly susceptible to dealumination.

**Figure 4:**
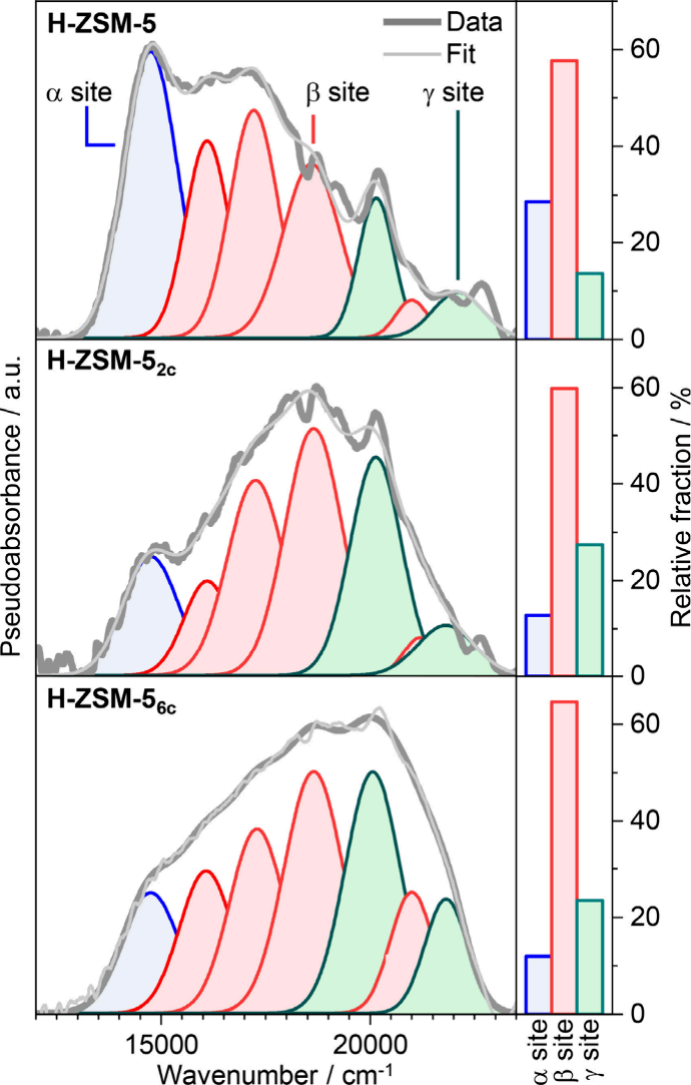
DR UV–vis spectra of dehydrated Co^2+^/Na^+^-ion-exchanged H-ZSM-5, H-ZSM-5_2c_, and H-ZSM-5_6c_ catalysts in the region of Co^2+^*d*–*d* transitions along with their deconvolution into site-specific Gaussian bends. The relative fraction of site-specific bands is shown on the right.

In summary, dealumination of H-ZSM-5 catalysts during recursive MTH reaction–regeneration cycles preferentially occurs at aluminum sites located in zeolite channels, with Al_F_ sites in straight channels being the most affected (Scheme 1). Isolated and proximate BAS sites are dealuminated with a similar propensity, which eventually results in a more pronounced relative loss of the latter with respect to the total BAS. The results provide important insights into the mechanism of zeolite dealumination by reaction-generated steam and demonstrate dynamic changes in acid site concentration and distribution in industrially relevant catalysts. The findings are also relevant for understanding the irreversible deactivation of zeolite catalysts and their rational engineering via steaming treatments.

## Supporting Information Available

Experimental information, catalytic data, ^27^Al, ^29^Si, ^1^H, ^1^H–^1^H, and ^27^Al–^27^Al DQ-SQ MAS NMR spectra (PDF)
