# Supplementary material for: Evidence of Preferential Aluminum Site Loss during Reaction-Induced Dealumination
Source: J Am Chem Soc. 2024 Dec 10;146(50):34279–84. doi: 10.1021/jacs.4c13212 (PMC11664574; doi:10.1021/jacs.4c13212)
Supplement: Supplementary file 1 — ja4c13212_si_001.pdf [file ja4c13212_si_001.pdf]

Supporting Information

Evidence of Preferential Aluminum Site Loss during Reaction-Induced Dealumination

Chao Wang,[1,2] Andreas Brenig,[1,3] Jun Xu,[2] Feng Deng,[2] Vladimir Paunović,[1,3,*] and Jeroen A. van Bokhoven[1,3,*]

[1]Institute for Chemical and Bioengineering, Department of Chemistry and Applied Biosciences, ETH Zurich, Vladimir-Prelog-Weg 1, 8093 Zurich, Switzerland

[2]National Center for Magnetic Resonance in Wuhan, State Key Laboratory of Magnetic Resonance and Atomic and Molecular Physics, Wuhan Institute of Physics and Mathematics, Innovation Academy for Precision Measurement Science and Technology, Chinese Academy of Sciences, Wuhan 430071, China

[3]Paul Scherrer Institute, Center for Energy and Environmental Sciences, PSI, 5232, Villigen, Switzerland

*E-mails: vladimir.paunovic@chem.ethz.ch

jeroen.vanbokhoven@chem.ethz.ch

S 1. Supporting Experimental Information

S.1.1. Catalyst and Dealumination Treatments. Commercial ZSM-5 zeolite (Zeolyst International) with Si/Al ratios of 39 (CBV 8014) was obtained in ammonium form. It was converted to a proton form by calcination under a flow of oxygen (PanGas, 5.0, F_{O2} = 100 cm^3_{STP} min^{-1}) at 823 K for 5 h using a heating rate of 2 K min^{-1}. The MTH conversion and steaming tests were carried out in an automated homemade continuous-flow fixed-bed reactor setup, described in our previous publication.[1] In the MTH reaction, the catalyst (catalyst weight, W_{cat} = 0.05 g, particle size, d_p = 180–250 μm) was mixed with quartz particles (Thommen-Furler, W_Q = 0.500 g, d_p = 350–500 μm) and loaded inside the quartz reactor. The catalyst bed was heated under argon flow (F_{Ar} = 300 cm^3_{STP} min^{-1}) to 823 K (heating rate of 10 K min^{-1}), then activated under oxygen (F_{O2} = 100 cm^3_{STP} min^{-1}) for 0.5 h, and finally cooled down to a desired reaction temperature under the argon flow. The MTH conversion was performed at 773 K and total pressure of P = 1.6 bar by using a methanol (CH_3OH Sigma Aldrich, HPLC grade, >99.9%) flow of F_{CH3OH} =0.08 cm^3_{lq} min^{-1} diluted with an argon flow of F_{Ar} = 190 cm^3_{STP} min^{-1}, which corresponds to a methanol concentration of c_{CH3OH} = 19 mol% in the feed and a weight-hourly space velocity of $WHSV$ = 76 h^{-1}. After a specific time leading to catalyst deactivation, the reaction was terminated by replacing the methanol feed with argon (F_{Ar} = 300 cm^3_{STP} min^{-1}), followed by purging of the reactor for 0.5 h. The argon flow was then replaced with an oxygen flow (PanGas, 5.0) of F_{O2} = 100 cm^3_{STP} min^{-1} and the catalyst bed was then heated to a temperature of 823 K (heating rate of 10 K min^{-1}) at which the regeneration was performed for 1 h. Thereafter, the oxygen flow was replaced with the flow of argon, and the regenerated catalyst bed was cooled down to a reaction temperature at which the next MTH reaction test was performed. The steaming of fresh catalyst was performed at 773 K and 1.6 bar using a flow rate of deionized (DI) water of F_{H2O} = 0.034 cm^3_{lq} min^{-1} and a flow of argon of F_{Ar} = 190 cm^3_{STP} min^{-1}, which resulted in a water concentration in the feed of c_{H2O} = 18 mol% and a weight-hourly space velocity of $WHSV$= 41 h^{-1}.

S.1.2. MAS NMR Experiments. The ^{27}Al MAS NMR spectra were acquired on Bruker Avance III HD 700 and Bruker Avance III 800 spectrometers, equipped with a 3.2 mm probe head at a spinning rate of 20 kHz, with resonance frequencies of 182.4 (700) and 208.52 (800) MHz for ^{27}Al nucleus. The ^{27}Al MAS NMR spectra were acquired using small-flip angle technique with a pulse length of 0.3 μs ($<\pi/12$) and a recycle delay of 1 s. The ^{27}Al chemical shifts were referenced to 1 M $Al(NO_3)_3$ aqueous solution (0 ppm). ^{29}Si MAS NMR spectra were measured on a Bruker Avance III HD 700 spectrometer equipped with a 3.2 mm probe head at a resonance frequency of 79.5 MHz using single-pulse excitation with a recycle delay of 5 s, a sweep width of 200 ppm, and 3072 accumulations. The magnetic field (and thereby the ppm axis) in ^{29}Si MAS NMR were calibrated using the ^{13}C MAS NMR spectrum of adamantane (at 38.52 ppm) as an external secondary standard. Solid-state ^{1}H MAS NMR (ssNMR) experiments were conducted on a Bruker Advance III 700 MHz spectrometer using a 3.2 mm probe

head and a spinning rate of 12 kHz. Adamantane was used as an external chemical shift reference for ^{1}H (δ 1.78) and ^{13}C (δ 38.5, -CH_2-), respectively. The samples were dehydrated at 723 K under vacuum at a residual pressure lower than 5×10^{-6} mbar for at least 12 h and loaded and sealed in the rotor inside the glovebox. Single-pulse ^{1}H MAS experiments were performed by using a π/2 pulse width of 3.8 μs and a repetition time of 2 s. All MAS NMR spectra were normalized with respect to the sample weight. The 2D ^{1}H double-quantum single-quantum (2D ^{1}H-^{1}H DQ-SQ) experiments were excited and reconverted with the back-to-back (BABA) pulse sequence with a spinning rate of 18 kHz.[2] DQ-SQ spectra were acquired by averaging 128 transients, respectively, with recycle delay of 1 s. The 2D ^{1}H-^{1}H DQ-SQ NMR spectrum was acquired with 128 t_1 increments of 55.56 μs at 1.053 ms recoupling time. Specifically, the 2D ^{1}H-^{1}H DQ-SQ correlation NMR spectra at different recoupling times were recorded at 9.4 T on a Bruker Avance III-400 spectrometer using commercial 3.2 mm rotors at a spinning rate of 12 kHz, with resonance frequencies of 399.33 MHz for ^{1}H. Double-quantum coherences were excited and reconverted with the $R12_5^2$ pulse sequence with $\nu_{^1H} = 3\nu_R = 36$ kHz, and the excitation and reconversion time were set to 0.33, 0.50 and 0.67 ms. The increment interval in the indirect dimension was set to 41.7 μs with a recycle delay of 2 s, and 128 t_1 increments, and 32 scans accumulations for each t_1 increment were used.

S.1.3. DR UV-vis Experiments. H-ZSM-5 catalyst and respective aged materials recovered after two (H-ZSM-5_{2c}) and six (H-ZSM-5_{6c}) consecutive MTH reaction and regeneration cycles were ion-exchanged three times using a 1 M sodium-nitrate ($NaNO_3$, Sigma-Aldrich, > 99%) solution of DI water (150 $cm^3_{solution}\ g_{zeolite}^{-1}$). Thereafter, they were ion-exchanged using a 0.05 M cobalt nitrate ($Co(NO_3)_2 \times 6H_2O$, Sigma-Aldrich, ≥ 99.9%) solution (150 $cm^3_{solution}\ g_{zeolite}^{-1}$). Every ion-exchange step was followed by washing with DI water. After completion of sodium and cobalt ion-exchanges, the samples were dried under vacuum (≤ 15 mbar) at 343 K for 24 h.

The Co/Na-exchanged ZSM-5 materials (*ca.* 45 mg) and H-ZSM-5 zeolite used for collecting the reference spectrum were pressed into self-supported pellets and loaded in a home-made quartz cell. The samples were dehydrated at 723 K (heating rate 15 K min^{-1}) in high vacuum ($P \sim 1\times10^{-10}$ bar) for 1 h, then calcined under oxygen atmosphere (P_{O2} = 0.52 bar) for 1 h, and evacuated again in high vacuum for 1 h. After cooling down to room temperature, the DR UV-Vis spectra were collected using a Ocean Optics DH-2000-BAL deuterium/halogen light source, a 200-1100 nm Ocean Optics six-around-one reflection probe, and an Ocean Optics Maya 2000-Pro UV-vis spectrometer. The parent H-ZSM-5 zeolite pellet was positioned below the Co/Na-exchanged ZSM-5 materials and used as the reference white standard. The final DR UV-Vis spectra were obtained by averaging *ca.* 300-350 spectra collected with a spectrometer integration time of *ca.* 30 ms. The region of the spectra sensitive for the Co-speciation (*ca.* 12000-25000 cm^{-1}) was deconvoluted in into seven Gaussian peaks using Origin(Pro) 2021b Software (OriginLab Corporation, Northampton, MA, USA) according to a previously reported

protocol.[3,4] The position of peaks and their corresponding full width at half maximum (FWHM) were restrained to a variation of ±150 cm^{-1} to ensure a minimal influence of peak position and shape on the relative contribution of various components in the spectra of different samples.

S.1.4. Hexane Cracking Experiments. Cracking of *n*-hexane (Fisher Scientific, 97%, Extra Dry) and 3-methylpentane (3MP, Acros Organics, >99%) was conducted over H-ZSM-5 and H-ZSM-5_{6c} catalysts using the same continuous flow fixed-bed reactor set-up employed for MTH-regeneration tests. Liquid alkane (F_{C6H14} = 0.009 cm^3_{lq} min^{-1}) was dosed by a syringe pump and evaporated into an argon carrier flow (F_{Ar} = 30 cm^3_{STP} min^{-1}) supplied by an MFC. To suppress cracking over surface-exposed acid sites, 2,4-dimethylquinoline (2,4-DMQ, Tokyo Chemical Industry, >95.0%) was added to the alkane feed (C_6H_{14}:2,4-DMQ = 500:1 mol mol^{-1}) The reaction was performed over a catalyst bed consisting of zeolite (W_{cat} = 0.006 g, d_p = 0.18-0.25 mm) well-mixed with quartz particles (W_Q = 0.200 g, d_p = 350–500 μm) at T = 751 K and P = 1.2 bar. These feed conditions correspond to a reactant concentration of c_{C6H14} = 4.8 mol% and WHSV = 16 h^{-1}.

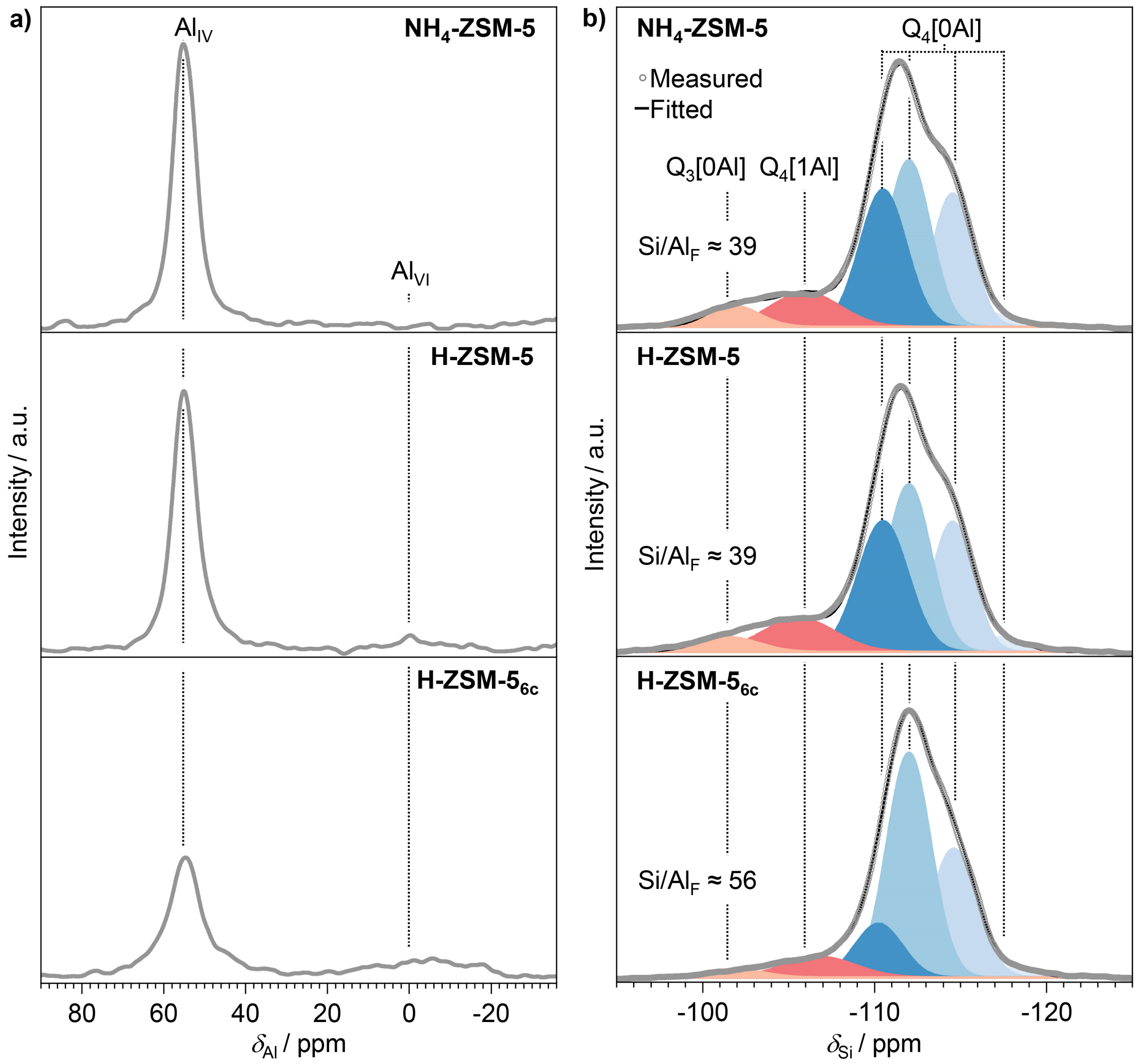

Figure S1. a) ^{27}Al and b) ^{29}Si MAS NMR spectra of NH_4-ZSM-5, H-ZSM-5, and H-ZSM-5$_{6c}$ catalysts. The ^{27}Al MAS NMR spectra of NH_4-ZSM-5 display a prominent tetrahedral aluminum signal (Al_{IV}, *ca*. 53-60 ppm) and virtually no detectable octahedral aluminum signal (Al_{VI}, *ca*. 0 ppm). In H-ZSM-5 zeolite the Al_{IV} signal decreases slightly (*ca*. 8% compared to NH_4-ZSM-5) in intensity, which is also accompanied by the appearance of the Al_{VI} resonance, indicating mild dealumination. A prominent decrease of the Al_{IV} signal intensity in H-ZSM-5$_{6c}$ zeolite (*ca*. 62% compared to NH_4-ZSM-5) indicates a substantial dealumination. These changes are also reflected in ^{29}Si MAS NMR spectra, which are deconvoluted into signals associated with silicon atoms with one nearest aluminum neighbor (Q_4[1Al], *ca*. -106.5 ppm), with only silicon atoms in their first shell (Q_4[0Al], *ca*. -111 to -120 ppm, **Figure 4b**), and defect Si sites (Q_3[0Al], ca. -102 ppm).[5–7] The estimated framework Si/Al ratio (Si/Al$_F$) is virtually not changing in H-ZSM-5 with respect to NH_4-ZSM-5 and is changing substantially in H-ZSM-5$_{6c}$ zeolite.

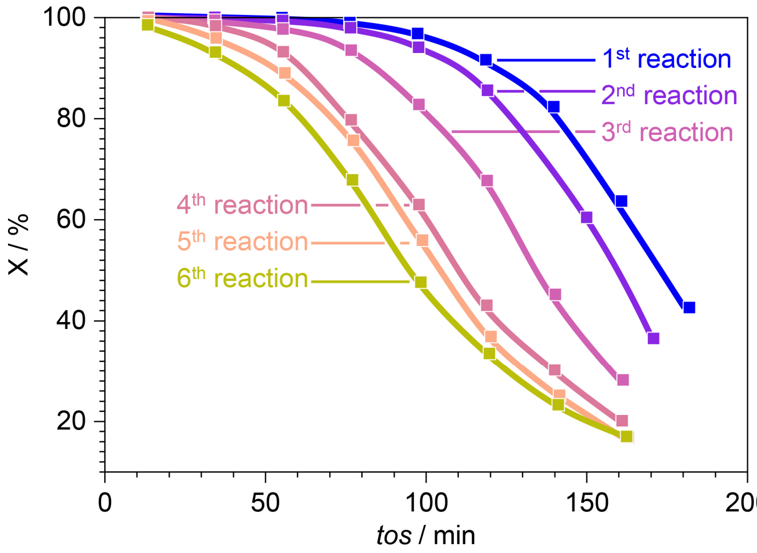

Figure S2. Conversion versus time-on-stream (*tos*) during sequential MTH reactions followed by the oxidative regeneration over H-ZSM-5 catalyst. MTH conditions: CH_3OH:Ar = 19:81 mol%, $WHSV = 76\ g_{CH3OH}\ g_{cat}^{-1}\ h^{-1}$, and $T = 773$ K. Regeneration conditions: 100 mol% O_2, $WHSV = 169\ g_{O2}\ g_{cat}^{-1}\ h^{-1}$, $T = 823$ K, and *tos* = 1 h. All tests were performed at $P = 1.6$ bar. The catalytic tests indicate a substantial decrease in the methanol cumulative turnover capacity after six successive reaction-regeneration runs, which points to a severe zeolite dealumination.

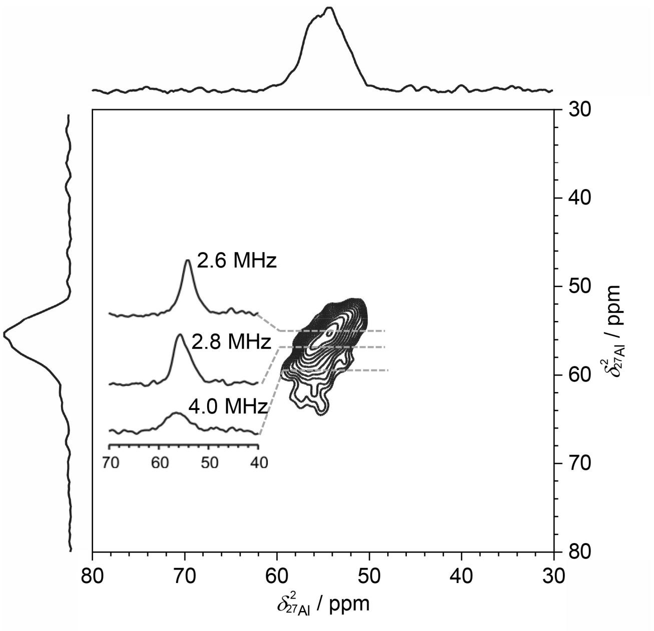

Figure S3. ^{27}Al 3Q MAS NMR spectra of H-ZSM-5 catalyst along with identified components used for deconvolution of 1D ^{27}Al MAS NMR spectra.

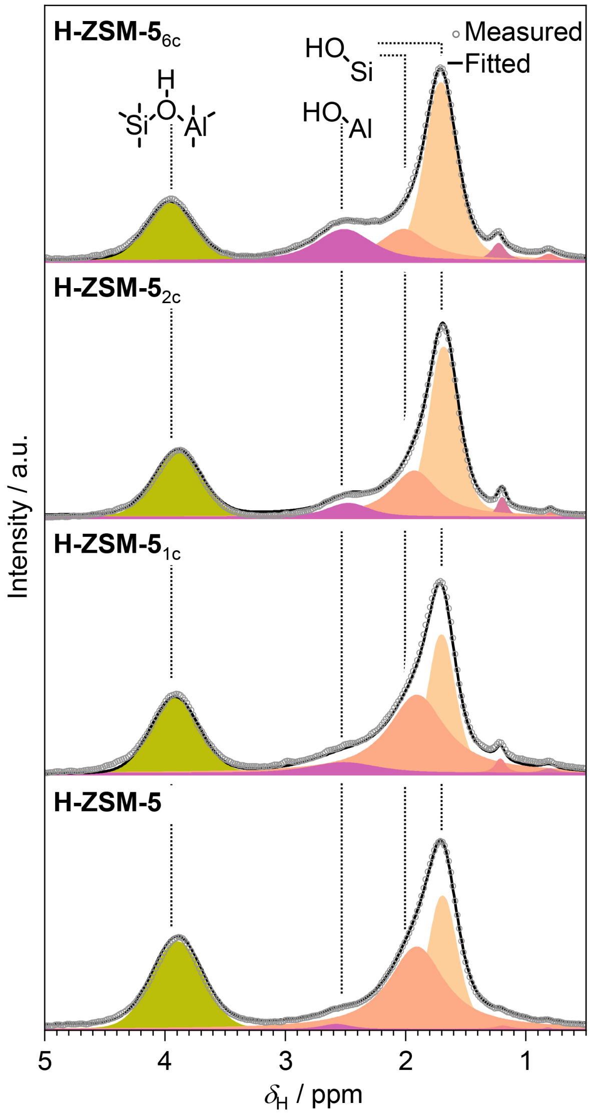

Figure S4. ^{1}H MAS NMR spectra of representative H-ZSM-5 catalysts in their fresh form and after operation in different numbers of successive MTH conversion-regeneration reactions presented in **Figure S2**. ^{1}H MAS NMR spectra are deconvoluted into the components ascribed to bridged aluminum sites, i.e., BAS (3.8 ppm), Al-OH groups (2.6 ppm) in Al_{FA} or Al_{EF} sites that likely exist as small aluminum oxide moieties, silanol Si-OH groups (1.6-2 ppm), and Al-OH groups of isolated Al_{EF} site potentially interacting with silanols (*ca.* 1.1-1.2 ppm and 0.8 ppm).[8–11] The spectra indicate a progressive decrease in BAS concentration and increase in Al-OH group concentration during sequential MTH reactions followed by the oxidative regeneration.

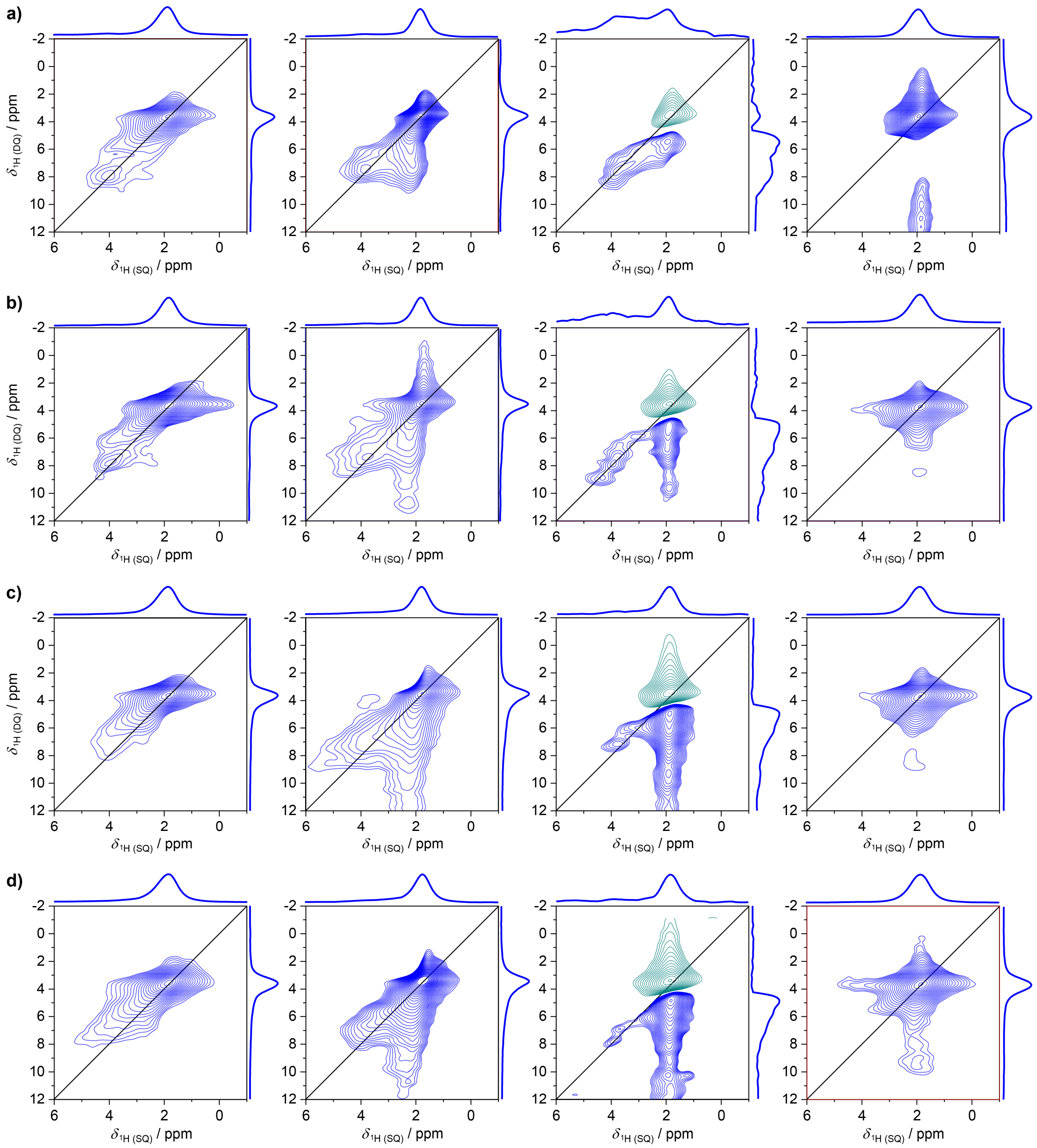

Figure S5. ^{1}H-^{1}H DQ-SQ MAS NMR spectra of **a)** H-ZSM-5, **b)** H-ZSM-5$_{1c}$, **c)** H-ZSM-5$_{2c}$, and **d)** H-ZSM-5$_{6c}$ catalysts collected at three different recoupling times of 0.33 (1st column), 0.5 (2nd column), and 0.66 (3rd and 4th column) ms. The spectra of fresh H-ZSM-5 display prominent Si-OH-Si-OH (1.9, 3.8 ppm), BAS-BAS (3.9, 7.8 ppm) autocorrelation as well as cross-correlation Si-OH-BAS (1.9, 5.8 ppm) peaks. The spectra indicate a pronounced decrease of the BAS-BAS autocorrelation signal with the progress of reaction-induced dealumination. The green lines in experiment performed at 0.66 ms represent the inversion signal due to the large recoupling time involved. Under large recoupling time,

it is challenging to adjust the phase for the BAS-BAS and SiOH-SiOH interactions simultaneously. The phase of BAS-BAS (3rd column) and Si-OH-Si-OH (4th column) signals are thus adjusted separately.

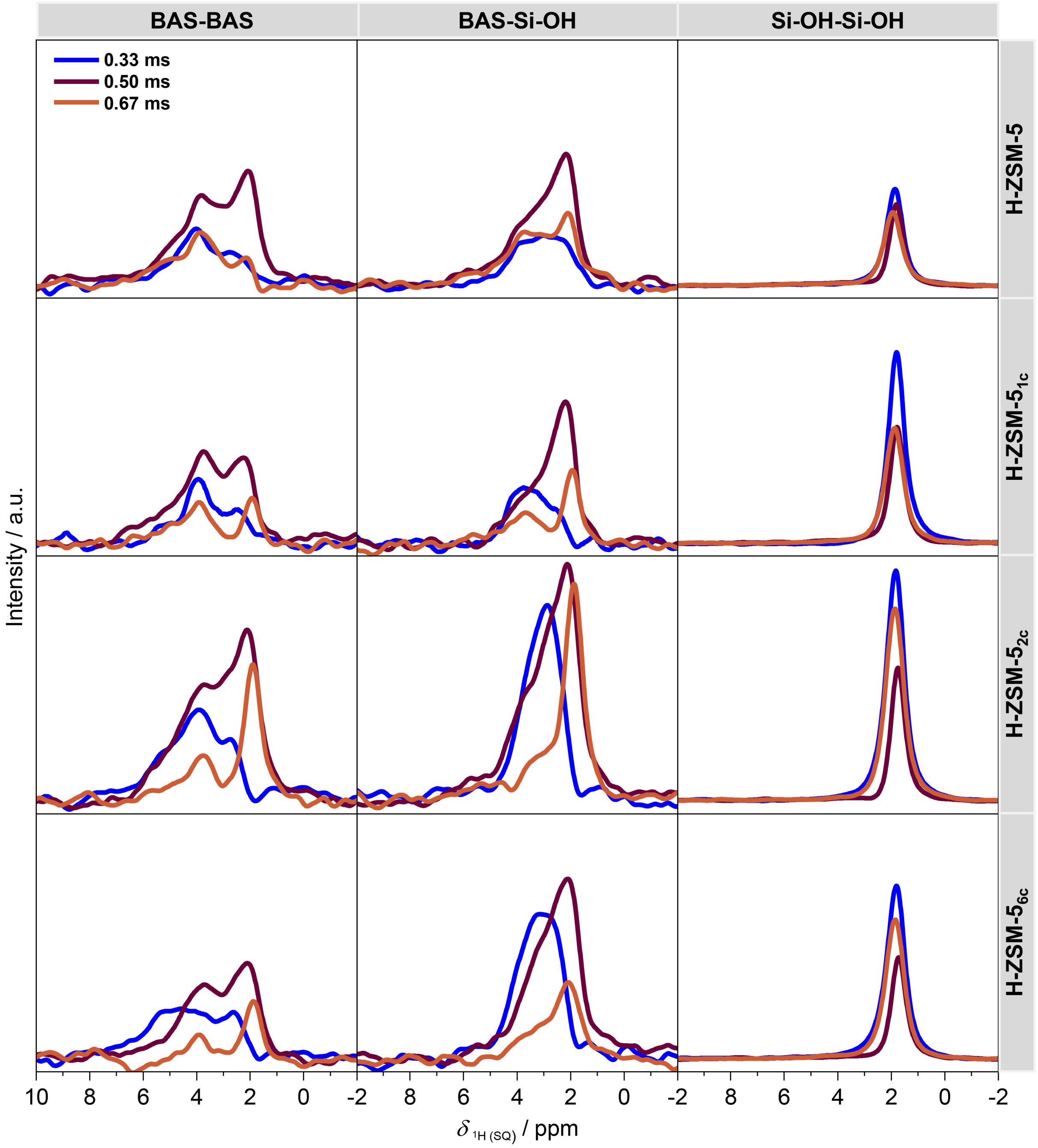

Figure S6. Slices along SQ dimension corresponding to BAS-BAS and Si-OH-Si-OH autocorrelation and BAS-Si-OH cross-correlation peak intensities as a function of recoupling time for H-ZSM-5, H-ZSM-5$_{1c}$, H-ZSM-5$_{2c}$, and H-ZSM-5$_{6c}$ catalysts. The slices were extracted from the ^1H-^1H DQ-SQ MAS NMR spectra presented in **Figure S5**.

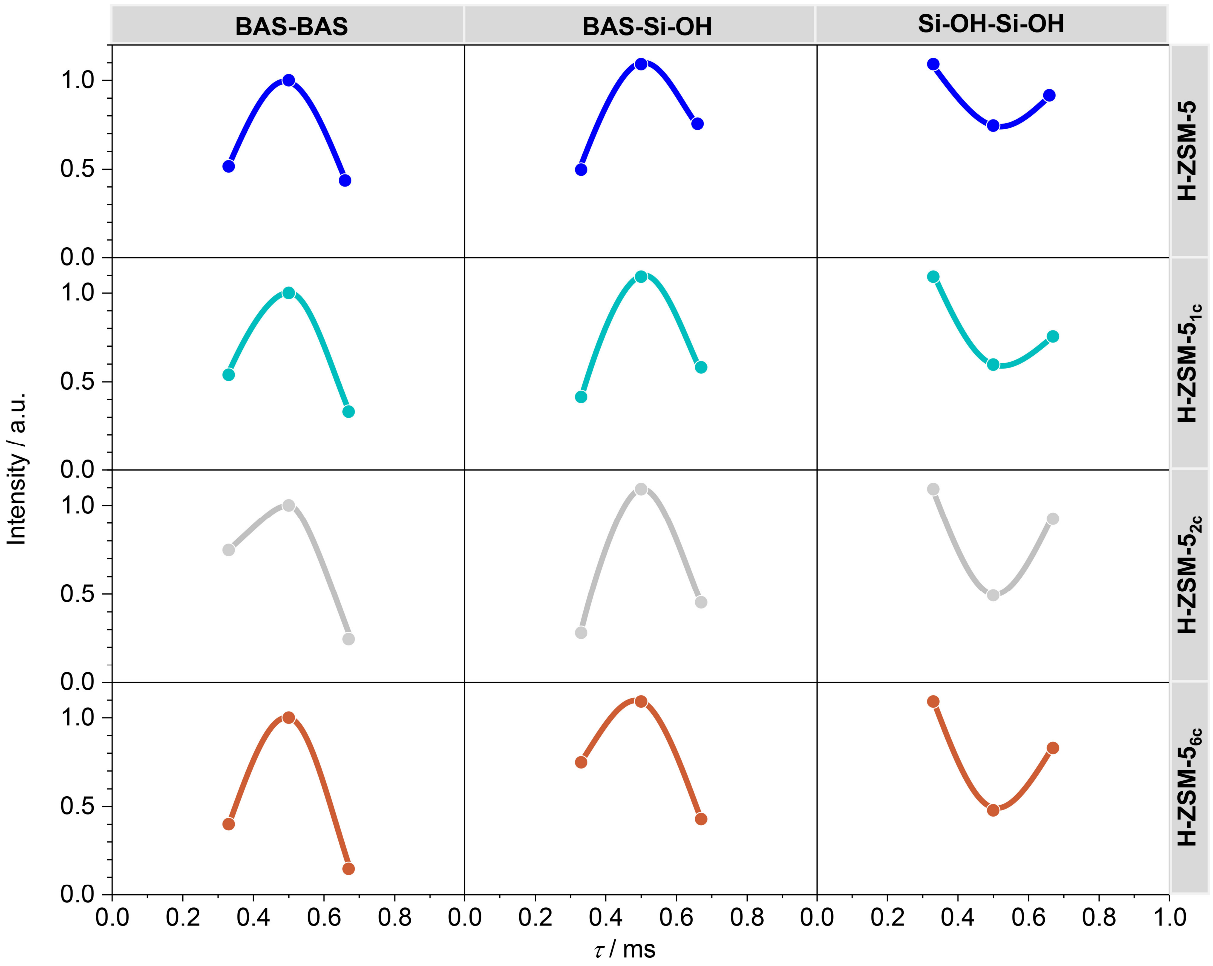

Figure S7. A comparison of BAS-BAS and Si-OH-Si-OH autocorrelation and BAS-Si-OH cross-correlation peak intensities as a function of recoupling time for H-ZSM-5, H-ZSM-5_{1c}, H-ZSM-5_{2c}, and H-ZSM-5_{6c} catalysts. The intensities are extracted from the slices presented in Figure S6, taking into account differences in δ_1 values and normalized with respect to the maximum intensity. The intensities of the autocorrelation and cross-correlation peaks as a function of recoupling times follow a rather similar trend indicating no significant changes in the distance between the proximate BAS sites.[12]

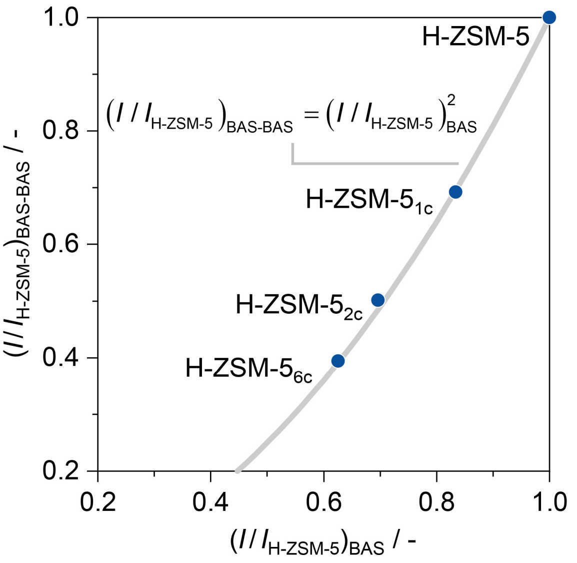

Figure S8. The relative decrease of the intensity of the BAS-BAS autocorrelation signal in 2D ^{1}H-^{1}H DQ-SQ MAS NMR spectra *versus* the relative decrease of the BAS peak in 1D ^{1}H MAS NMR spectra (**Figure S4**) in corresponding catalysts with respect to those in H-ZSM-5 zeolite. Considering the unit cell formula of H-ZSM-5 zeolite (**MFI** topology), $H_xAl_xSi_{96-x}O_{192}$, it follows that the material under investigation contains 2.4 Al_F and 93.4 Si_F sites per unit cell on average. Assuming a random (normal) distribution of Al_F atoms and hence BAS across various T site positions, it follows that the sample is mostly composed of unit cells comprising 2 or 3 Al_F sites per unit cell. Both isolated and proximate BAS are reflected in a resonance centered at around 3.8 ppm in 1D ^{1}H MAS NMR spectrum (Figure 1), the intensity of which is directly proportional to the concentration of these sites (**Eq. S1**).

$$I_{\text{BAS}} \propto c_{\text{BAS}} \qquad \textbf{Eq. S1}$$

Taking into account the typical dimensions of the orthorhombic **MFI** unit cell ($a \times b \times c$ = 20.1×19.7 ×13.1 Å^3), an average BAS is found in the volume range of *ca.* 1730 (3 Al_F unit cell^{-1}) - 2590 (2 Al_F unit cell^{-1}) Å^{-3}. It is further assumed that the BAS-BAS autocorrelation peak arises from the protons that are at spatial distance $r_d \approx 5$ Å or less,[12] which corresponds to a BAS concentration of *ca.* 170 Å^{-3}. Considering low Al_F concentration, it can be assumed that the proximate sites primarily comprise proximate BAS-BAS pairs, while the configurations with three of more proximate BAS sites are virtually absent. The intensity of the autocorrelation peak is further considered proportional to the concentration of proximate sites, which under the assumption of random dealumination model and following the premises analogues to the collision theory is proportional to the square of BAS concentration (**Eq. S2**).

$$I_{\text{BAS-BAS}} \propto c_{\text{BAS}} \times c_{\text{BAS}} \qquad \textbf{Eq. S2}$$

Based on these assumptions, the relative change of $I_{\text{BAS-BAS}}$ in dealuminated with respect to parent zeolite with initial BAS concentration, $c_{\text{BAS,0}}$, (**Eq. S3,4**) is expected to approximately follow the second-order dependence with respect to the relative change of BAS resonance intensity (**Eq. S5**).

$$\left(\frac{I}{I_{\text{H-ZSM-5}}}\right)_{\text{BAS}} \propto \frac{c_{\text{BAS}}}{c_{\text{BAS, H-ZSM-5}}} \qquad \textbf{Eq. S3}$$

$$\left(\frac{I}{I_{\text{H-ZSM-5}}}\right)_{\text{BAS-BAS}} \propto \frac{c_{\text{BAS}} \times c_{\text{BAS}}}{c_{\text{BAS, H-ZSM-5}} \times c_{\text{BAS, H-ZSM-5}}} \qquad \textbf{Eq. S4}$$

$$\left(\frac{I}{I_{\text{H-ZSM-5}}}\right)_{\text{BAS-BAS}} = \left(\frac{I}{I_{\text{H-ZSM-5}}}\right)^{2}_{\text{BAS}} \qquad \textbf{Eq. S5}$$

The outcome of these considerations indicates that the relative concentration of proximate BAS sites, and consequently the associated autocorrelation signal, is expected to decrease more significantly than the relative change in total BAS concentration and the corresponding resonance observed in 1D ^{1}H MAS NMR spectra, even under conditions of random dealumination. Notably, the experimental values of relative changes in autocorrelation and BAS resonances show good agreement with the predicted second-order dependence between the signal intensities (gray line, **Figure S8**). This indicates that dealumination of both single and proximate sites occurs with a similar preference. Still, due to statistical reason arising from the fact that dealumination of any of the proximate BAS-BAS sites leads to a loss of the proximate pair, the concentration of proximate sites is much more affected by the dealumination process.

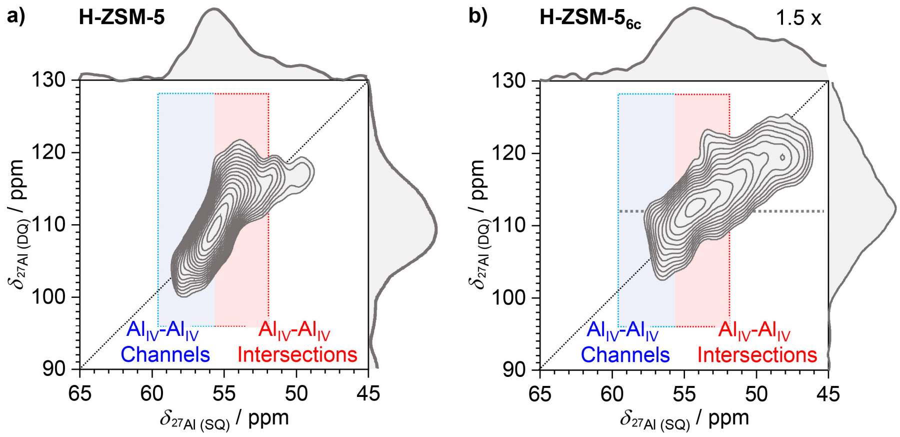

Figure S9. 2D ^{27}Al-^{27}Al DQ-SQ MAS NMR spectra (16.4 T) of **a)** H-ZSM-5 and **b)** H-ZSM-5_{6c} catalysts in the region of the tetrahedral Al_F sites resonances. The blue and red shaded areas in a,b) indicate the regions of the proximate Al_F sites located in channels and their intersections, respectively.

Additional information on the dealumination processes is extracted from the 2D ^{27}Al-^{27}Al double quantum (DQ)-single quantum (SQ) MAS NMR spectroscopy (**Figure S9**).[13–15] In respective 2D ^{27}Al-^{27}Al DQ-SQ MAS NMR spectra, the autocorrelation peak (δ_i, 2×δ_i), positioned along the spectral diagonal indicates the spatial proximity of homogeneous spin pairs, while the intercorrelation peak (δ_i, δ_i+δ_j) indicates the formation of heterogenous *j-i.* spin pairs. In view of quantum effects exploited by the DQ-SQ MAS NMR sequence, the correlation peaks indicate the interaction between the aluminum sites within spatial distances of *ca.* 5 Å. The spectra of H-ZSM-5 exhibit the clear autocorrelation signal that is centered at *ca.* (55, 110) ppm in the range of Al_{IV} resonances, corroborating the presence of spatially proximate Al_F sites. Based on considerations made for the ^{27}Al and 1D and 3Q MAS spectra, this signal can be divided into the zones associated with proximate Al_F sites in the channels ($\delta_{27Al(SQ)} \approx$ 55.5-59 ppm) and intersections ($\delta_{27Al(SQ)} \approx$ 52-55.5 ppm), along with some proximate non-framework Al sites ($\delta_{27Al(SQ)} \leq$ 52 ppm). Importantly, the signal arising from the silicon T sites with two aluminum atoms in their second coordination sphere is below the detection limit in ^{29}Si MAS NMR spectra of the H-ZSM-5 zeolite, suggesting a very low concentration of paired aluminum sites sharing the same silicon tetrahedra, (*i.e.*, Al-O-Si-O-Al, **Figure S1**). This indicates that the autocorrelation peak in the 2D ^{27}Al-^{27}Al DQ-SQ spectra mostly arises from the spatially close aluminum T sites that are sufficiently distant across the zeolite channels. Although the ^{27}Al-^{27}Al DQ-SQ autocorrelation signal is not absolutely quantitative, partially because of the potential presence of unresolved aluminum sites (*e.g.*, with somewhat larger distance and smaller dipolar coupling constant) that cause the autocorrelations not to be visible along the diagonal,[16], the intensity of the Al_{IV} signals in the 2D ^{27}Al-^{27}Al DQ-SQ MAS NMR spectra of H-ZSM-5_{6c} decreased as compared to H-ZSM-5. In addition, the signal in these spectra decreased much more prominently in the higher-frequency region centered of *ca.* 56 ppm, indicating that proximate Al_F sites in the channels are more prone to dealuminate ones in their intersections. These changes are consistent with those in respective ^{27}Al 3Q MAS NMR and 1D MAS NMR spectra.

Supporting Information References

(1) Paunović, V.; Hemberger, P.; Bodi, A.; Hauert, R.; van Bokhoven, J. A. Impact of Nonzeolite-Catalyzed Formation of Formaldehyde on the Methanol-to-Hydrocarbons Conversion. *ACS Catal.* **2022**, *12*, 13426–13434. https://doi.org/10.1021/acscatal.2c02953.

(2) Zheng, M.; Zeng, S.; Wang, X.; Gao, X.; Wang, Q.; Xu, J.; Deng, F. Heteronuclear-Filtered ^{1}H Homonuclear Multi-Quantum Correlation Experiment at 100 KHz Magic-Angle Spinning. *Magn. Reson. Lett.* **2022**, *2*, 266–275. https://doi.org/10.1016/j.mrl.2022.09.002.

(3) Dědeček, J.; Balgová, V.; Pashkova, V.; Klein, P.; Wichterlová, B. Synthesis of ZSM-5 Zeolites with Defined Distribution of Al Atoms in the Framework and Multinuclear MAS NMR Analysis of the Control of Al Distribution. *Chem. Mater.* **2012**, *24*, 3231–3239. https://doi.org/10.1021/cm301629a.

(4) Dědeček, J.; Kaucký, D.; Wichterlová, B.; Gonsiorová, O. Co^{2+} Ions as Probes of Al Distribution in the Framework of Zeolites. ZSM-5 Study. *Phys. Chem. Chem. Phys.* **2002**, *4*, 5406–5413. https://doi.org/10.1039/b203966b.

(5) Liang, T.; Chen, J.; Qin, Z.; Li, J.; Wang, P.; Wang, S.; Wang, G.; Dong, M.; Fan, W.; Wang, J. Conversion of Methanol to Olefins over H-ZSM-5 Zeolite: Reaction Pathway Is Related to the Framework Aluminum Siting. *ACS Catal.* **2016**, *6*, 7311–7325. https://doi.org/10.1021/acscatal.6b01771.

(6) He, L.-H.; Li, J.-J.; Han, S.-Y.; Fan, D.; Li, X.-J.; Xu, S.-T.; Wei, Y.-X.; Liu, Z.-M. Dynamic Evolution of HZSM-5 Zeolite Framework under Steam Treatment. *Chem. Synth.* **2023**, *3*, 1–18. https://doi.org/10.20517/cs.2023.55.

(7) Holzinger, J.; Beato, P.; Lundegaard, L. F.; Skibsted, J. Distribution of Aluminum over the Tetrahedral Sites in ZSM-5 Zeolites and Their Evolution after Steam Treatment. *J. Phys. Chem. C* **2018**, *122*, 15595–15613. https://doi.org/10.1021/acs.jpcc.8b05277.

(8) Treps, L.; Demaret, C.; Wisser, D.; Harbuzaru, B.; Méthivier, A.; Guillon, E.; Benedis, D. V.; Gomez, A.; Bruin, T. De; Rivallan, M.; Catita, L.; Lesage, A.; Chizallet, C. Spectroscopic Expression

of the External Surface Sites of H-ZSM-5. *J. Phys. Chem. C* **2021**, *125*, 2163–2181. https://doi.org/10.1021/acs.jpcc.0c10200.

(9) Zhao, Z.; Xiao, D.; Chen, K.; Wang, R.; Liang, L.; Liu, Z.; Hung, I.; Gan, Z.; Hou, G. Nature of Five-Coordinated Al in γ-Al_2O_3 Revealed by Ultra-High-Field Solid-State NMR. *ACS Cent. Sci.* **2022**, *8*, 796–803. https://doi.org/10.1021/acscentsci.1c01497.

(10) Chen, K.; Abdolrhamani, M.; Sheets, E.; Freeman, J.; Ward, G.; White, J. L. Direct Detection of Multiple Acidic Proton Sites in Zeolite HZSM-5. *J. Am. Chem. Soc.* **2017**, *139*, 18698–18704. https://doi.org/10.1021/jacs.7b10940.

(11) Ong, L. H.; Dömök, M.; Olindo, R.; Van Veen, A. C.; Lercher, J. A. Dealumination of HZSM-5 via Steam-Treatment. *Micropor. Mesopor. Mater.* **2012**, *164*, 9–20. https://doi.org/10.1016/j.micromeso.2012.07.033.

(12) Li, S.; Huang, S.-J.; Shen, W.; Zhang, H.; Fang, H.; Zheng, A.; Liu, S.-B.; Deng, F. Probing the Spatial Proximities among Acid Sites in Dealuminated H-Y Zeolite by Solid-State NMR Spectroscopy. *J. Phys. Chem. C* **2008**, *112*, 14486–14494. https://doi.org/10.1021/jp803494n.

(13) Yu, Z.; Li, S.; Wang, Q.; Zheng, A.; Jun, X.; Chen, L.; Deng, F. Brønsted/Lewis Acid Synergy in H-ZSM-5 and H-MOR Zeolites Studied by ^{1}H and ^{27}Al DQ-MAS Solid-State NMR Spectroscopy. *J. Phys. Chem. C* **2011**, *115*, 22320–22327. https://doi.org/10.1021/jp203923z.

(14) Yu, Z.; Zheng, A.; Wang, Q.; Chen, L.; Xu, J.; Amoureux, J.; Deng, F. Insights into the Dealumination of Zeolite HY Revealed by Sensitivity-Enhanced ^{27}Al DQ-MAS NMR Spectroscopy at High Field. *Angew. Chem. Int. Ed.* **2010**, *49*, 8657–8661. https://doi.org/10.1002/anie.201004007.

(15) Lee, D.; Takahashi, H.; Thankamony, A. S. L.; Dacquin, J. P.; Bardet, M.; Lafon, O.; Paëpe, G. De. Enhanced Solid-State NMR Correlation Spectroscopy of Quadrupolar Nuclei Using Dynamic Nuclear Polarization. *J. Am. Chem. Soc.* **2012**, *134*, 18491–18494. https://doi.org/10.1021/ja307755t.

(16) Gómez, J. S.; Trébosc, J.; Tuan Duong, N.; Pourpoint, F.; Lafon, O.; Amoureux, J.-P. Comparison of Through-Space Homonuclear Correlations between Quadrupolar Nuclei in Solids. *J. Magn. Reson.* **2023**, *348*, 107388. https://doi.org/10.1016/j.jmr.2023.107388.
